# When to add anti-angiogenesis drugs to EGFR-mutated metastatic non–small cell lung cancer patients: a real-world study from Taiwan

**DOI:** 10.1186/s12885-022-09672-4

**Published:** 2022-05-23

**Authors:** Chieh-Lung Chen, Sing-Ting Wang, Wei-Chih Liao, Chia-Hung Chen, Chih-Yen Tu, Hung-Jen Chen, Te-Chun Hsia, Wen-Chien Cheng

**Affiliations:** 1 Division of Pulmonary and Critical Care, Department of Internal Medicine, China Medical University Hospital, No. 2, Yude Road, North District, Taichung City, 40402 Taiwan; 2 Division of Hematology and Oncology, Department of Internal Medicine, China Medical University Hospital, No. 2, Yude Road, North District, Taichung City, 40402 Taiwan; 3 School of Medicine, College of Medicine, China Medical University, No. 91, Xueshi Road, North District, Taichung City, 40402 Taiwan; 4 Department of Life Science, National Chung Hsing University, No. 145, Xingda Road, South District, Taichung City, 402 Taiwan; 5 Ph.D. Program in Translational Medicine, National Chung Hsing University, No. 145, Xingda Road, South District, Taichung City, 402 Taiwan; 6 Rong Hsing Research Center for Translational Medicine, National Chung Hsing University, No. 145, Xingda Road, South District, Taichung City, 402 Taiwan

**Keywords:** Non–small cell lung cancer (NSCLC), Epidermal growth factor receptor (EGFR), Anti-angiogenesis

## Abstract

**Background:**

The addition of anti-angiogenesis drugs to epidermal growth factor receptor (EGFR)–tyrosine kinase inhibitor (TKI) or chemotherapy in patients with EGFR-mutant non–small cell lung cancer (NSCLC) can improve disease control. We conducted a study to evaluate the efficacy of combination therapeutic strategies and identify patients who could benefit from combination therapy.

**Methods:**

This study enrolled patients with stage IV EGFR-mutant NSCLC treated with first-line EGFR-TKIs between January 2014 and December 2020. We divided patients into three groups: patients who received an anti-angiogenesis drug as first-line combination therapy, those who received an anti-angiogenesis drug as further-line combination therapy, and those with no anti-angiogenesis therapy.

**Results:**

A total of 204 patients were enrolled in the final analysis. Progression-free survival (PFS) in patients receiving first-line anti-angiogenesis plus EGFR-TKI combination therapy was longer (18.2 months) than those treated with first-line EGFR-TKI monotherapy (10.0 months for both, *p* < 0.001). No difference in overall survival (OS) was observed among these three groups (30.5 vs. 42.6 vs. 33.7 months, *p* = 0.326). Multivariate Cox regression analysis revealed L858R mutation, pleural, liver, and bone metastasis as independent prognostic factors for poor OS. However, the addition of anti-angiogenesis therapy to patients with these poor prognostic factors improved OS to levels similar to those without these poor prognostic factors.

**Conclusion:**

First-line combination EGFR-TKI plus anti-angiogenesis therapy improves PFS in patients with stage IV EGFR-mutant NSCLC. Adding an anti-angiogenesis drug at any line to patients harboring L858R mutation with pleural, liver, or bone metastases can provide survival benefits.

## Background

Epidermal growth factor receptor (EGFR) tyrosine kinase inhibitors (TKI), from first-generation to third-generation agents, have revolutionized therapeutic strategies for advanced-stage non–small cell lung cancer (NSCLC) harboring EGFR mutations, improving the quality of life and outcomes of patients [1–6]. Afatinib and dacomitinib, second-generation EGFR-TKIs, form irreversible covalent bonds with the tyrosine kinase domain in pan-ErbB receptors [3, 4] and display remarkable efficacy and longer progression-free survival (PFS) than first-generation EGFR-TKIs [4, 5]. Osimertinib, a third-generation EGFR-TKI, also displays superior efficacy compared with first-generation EGFR-TKIs when used as first-line treatment [6]. Most patients treated with first- or second-generation EGFR-TKIs eventually develop acquired resistance, most commonly due to the EGFR T790M mutation [7]. In the AURA3 study, sequential first- and second-generation EGFR-TKI and osimertinib demonstrated significantly longer PFS and a better objective response rate (ORR) among patients with NSCLC harboring the EGFR T790M mutation [8]. However, suitable therapy options following acquired resistance to osimertinib remain a challenge [9]. Therefore, a debate exists regarding the optimal therapeutic strategy to achieve the best survival outcome: first-line osimertinib or sequential therapy consisting of either a first- or second-generation EGFR-TKI, followed by osimertinib. The sequence of treatments has become an important issue for improving treatment efficacy and prolonging the duration of first-line therapy.

Several studies have reported crosstalk between EGFR and vascular endothelial growth factor (VEGF) and its receptor (VEGFR), and promising synergic effects have been described by targeting both pathways [10–12]. The efficacy of combination treatment with an anti-angiogenesis drug and an EGFR-TKI has been evaluated in many randomized controlled clinical trials [13–15]. The JO25567 phase II study showed improved PFS for erlotinib plus bevacizumab compared with erlotinib alone (16 months vs. 9.7 months; *p* = 0.0015) [13]. NEJ026 and RELAY phase III studies also reported significantly longer PFS for erlotinib combined with either bevacizumab (16.9 months vs. 13.3 months; *p* = 0.016) or ramucirumab (19.4 months vs. 12.4 months; *p* < 0.001), respectively, compared with erlotinib alone [14, 15]. Most trials have been limited to populations treated with first-line erlotinib, and additional studies remain necessary to evaluate the efficacy of other EGFR-TKIs in combination with either bevacizumab or ramucirumab. Recently, a few real-world studies demonstrated the treatment efficacy of these combinations. Huang et al. demonstrated that different EGFR-TKIs (erlotinib and afatinib) plus bevacizumab provided similar clinical efficacy for treatment of advanced-stage EGFR-mutant lung adenocarcinoma [16]. A multicenter observational study reported that the combination of afatinib and bevacizumab provided positive clinical outcomes with acceptable safety profiles in untreated advanced-stage, EGFR-mutant NSCLC [17].

Although most clinical trials have shown significant benefits for PFS, few have reported significant benefits in overall survival (OS), which may be explained by the crossover rate, not including OS as a primary endpoint in the study design, a lack of standard protocol of care after disease progression, and the criteria used for patient selection. The REVEL study showed that VEGF pathway inhibition coupled with second-line chemotherapy had a larger effect in patients with advanced NSCLC [18]. Another study reported that the addition of bevacizumab could be a useful therapeutic strategy for progression in patients with EGFR-mutant NSCLC following EGFR-TKI failure [19]. Progression-free survival 2 (PFS-2), defined as the time from randomization to progression on second-line therapy, was moderately correlated with OS [20]. Therefore, whether the combination of an EGFR-TKI plus an anti-angiogenesis agent as first-line treatment is able to improve OS for patients with EGFR-mutant NSCLC remains controversial. Recently, Tsai et al. indicated that the combination of an EGFR-TKI and bevacizumab not only improves PFS but also improves OS in patients with advanced-stage EGFR-mutant NSCLC harboring the L858R mutation [21]. Determining which patient groups may benefit from combination treatment and when it should be administered is crucial, as the response to treatment can be heterogeneous.

We performed a retrospective study to investigate which groups of patients with advanced-stage EGFR-mutant NSCLC are likely to derive a benefit in OS when treated with combination EGFR-TKI and anti-angiogenesis treatment. We also compared the clinical outcomes among patients treated at our practice between those who received first-line and further-line anti-angiogenesis combination therapy and compared combination treatment with treatment without the use of any anti-angiogenesis agents to clarify the optimal timing of adding anti-angiogenesis therapy.

## Materials and Methods

### Patient eligibility

This retrospective study was conducted to analyze patients with advanced-stage EGFR-mutant NSCLC treated with a first- or second-generation EGFR-TKI (gefitinib, erlotinib, or afatinib) as first-line therapy, with or without an anti-angiogenesis drug (bevacizumab or ramucirumab), at a tertiary referral center in Taiwan between January 2014 and December 2020. Patients who did not undergo re-biopsy after disease progression, those with stage IIIB/IIIC disease, those who experienced disease recurrence after resection, and those with insufficient data were excluded from the analysis. Only stage IV patients (according to American Joint Committee on Cancer, 8th edition) were enrolled in the final analysis. The study protocol was approved by the institutional ethics committee of the relevant institution (IRB number: CMUH110-REC1–244), and informed consent was waived due to the observational and retrospective study design. Data regarding the baseline characteristics of each patient, including age, sex, smoking status, Eastern Cooperative Oncology Group Performance Status (ECOG PS), tumor–node–metastasis (TNM) stage at initial diagnosis, the pattern of distant metastases, EGFR mutation subtype, first-line treatment, T790M status after disease progression, and subsequent antineoplastic therapy after disease progression, were also recorded.

Anti-angiogenesis therapy.

In this study, patients treated with the anti-angiogenesis agent bevacizumab received either 7.5 or 15 mg/kg body weight every 4 weeks; bevacizumab is not covered by the National Health Insurance program for lung cancer therapy in Taiwan, and 7.5 mg/kg body weight was demonstrated to be as effective as 15 mg/kg body weight when used in combination with chemotherapy [22]. Ramucirumab was administered at a dose of 8 mg/kg every 2 weeks according to the randomized control trial protocol [15]. All patients enrolled in the current study received at least 3 cycles of bevacizumab or ramucirumab, regardless of whether combination treatment including an anti-angiogenesis agent was used as a first-line therapy or a further-line treatment. First-line therapy was defined as the first systemic therapy after diagnosis of stage IV NSCLC. Further-line treatment was defined as any subsequent anti-cancer therapy administered after disease progression on any line of treatment.

### Clinical assessments and efficacy evaluations

At baseline, patients underwent an imaging study that included a chest computed tomography (CT), a brain CT or magnetic resonance imaging (MRI) in cases with neurologic signs, and positron emission tomography (PET) to determine the disease stage and evaluate metastasis. After initiation of EGFR-TKI therapy, all patients underwent chest CT every 12 weeks to evaluate tumor response. Other images were obtained and evaluated by a clinical physician. PFS was calculated from the time of EGFR-TKI therapy initiation until radiological progression (according to the Response Evaluation Criteria in Solid Tumors v1.1) or death. OS was calculated from EGFR-TKI therapy initiation until death. If patients were still alive on 21 October 2021, which was the last follow-up time point, survival was censored at the end of the study.

### Statistical analyses

All statistical analyses were analyzed using MedCalc for Windows version 18.10 (MedCalc Software, Ostend, Belgium). Data are expressed as the mean ± standard deviation or as the median and interquartile range (IQR) for variables with and without a normal distribution, respectively. Continuous data with normal distributions were analyzed using a t-test. For ordinal data and data that were not normally distributed, differences between groups were assessed using a Kruskal–Wallis test. Categorical variables were presented as the number and percentage and were analyzed using the Chi-square test. PFS and OS were evaluated using the Kaplan–Meier method. Prognostic factors were analyzed using the Cox proportional hazards regression analysis. A univariate analysis was used to calculate the hazard ratio (HR) for mortality. Significant variables on univariate analysis and clinically important variables were included in the multivariate regression model. The strength of association was presented as the HR and associated 95% confidence interval (CI). A *p*-value of < 0.05 was considered significant.

## Results

### Patients and clinical characteristics

**Fig. 1:**
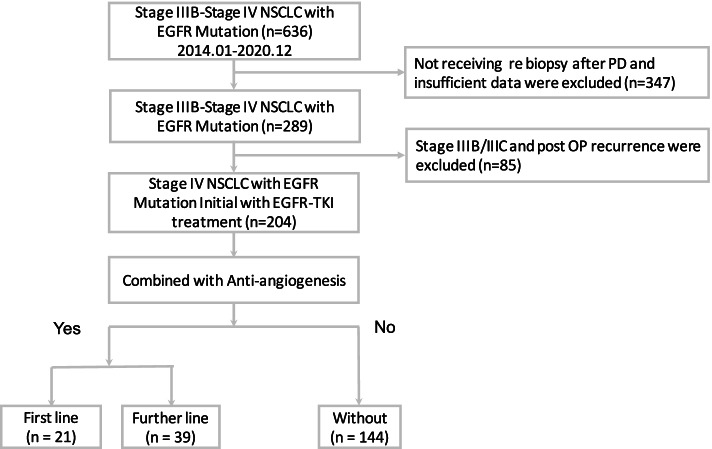
Flowchart for patient enrollment in the study. EGFR, epidermal growth factor receptor; TKI, tyrosine kinase inhibitor; NSCLC, non–small cell lung cancer; OP, operation; PD, progressive disease

A total of 204 patients with stage IV NSCLC receiving first-line EGFR-TKI therapy were enrolled in this study. Of these patients, 60 (29.4%) patients received combination therapy with an anti-angiogenesis drug, and 144 (70.6%) patients did not receive anti-angiogenesis therapy. Among those receiving anti-angiogenesis therapy, 21 patients underwent combination therapy with an anti-angiogenesis drug as first-line treatment, and 39 (19.1%) patients received an anti-angiogenesis drug as further-line combination therapy after disease progression (Fig. 1). The baseline characteristics of these patients are listed in Table 1. We identified 107 (52.5%) patients with EGFR exon 19 deletion and 88 (43.1%) patients with exon 21 L858R point mutation. Patients who received first- or further-line anti-angiogenesis therapy were significantly younger (*p* = 0.018) than those treated without anti-angiogenesis therapy. The most commonly used EGFR-TKIs were erlotinib (37.7%) and afatinib (37.3%), and erlotinib (52.4%) was mostly used in combination with first-line anti-angiogenesis therapy. In addition, 39 patients (19.1%) received bevacizumab, and 21 patients (10.3%) received ramucirumab. No significant differences in ECOG PS, smoking status, or the pattern of distant metastasis were observed between the three groups (Table 1).

**Table 1:** Patient Characteristics

	All (***n*** = 204)	With Anti-Angiogenesis (***n*** = 60)		Without Anti-Angiogenesis (***n*** = 144)	***p***-value
		First line (***n*** = 21)	Further line (***n*** = 39)		
**Age ≥ 65 years**	75 (36.8)	7 (33.3)	7 (17.9)	61 (42.4)	0.018
**Male**	68 (33.3)	8 (38.1)	14 (35.9)	46 (31.9)	0.796
**Smoking**	41 (20.1)	4 (19.0)	8 (20.5)	29 (20.1)	0.990
**ECOG PS** **≥** **2**	16 (7.8)	1 (4.8)	2 (5.1)	13 (9.0)	0.621
***EGFR*** **mutation**					0.086
**Del 19**	107 (52.5)	8 (38.1)	24 (61.5)	75 (52.1)	
**L858R**	88 (43.1)	10 (47.6)	15 (38.5)	63 (43.7)	
**Uncommon**	9 (4.4)	3 (14.3)	0 (0)	6 (4.2)	
**Metastasis Organ**					
**Lung metastasis**	102 (50.0)	10 (47.6)	20 (51.3)	72 (50.0)	0.964
**LN metastasis**	141 (69.1)	13 (61.9)	26 (66.7)	102 (70.8)	0.663
**Pleural metastasis**	92 (45.1)	5 (23.8)	16 (41.0)	71 (49.3)	0.076
**Liver metastasis**	40 (19.6)	5 (23.8)	9 (23.1)	26 (18.1)	0.686
**Bone Metastasis**	106 (52)	14 (66.7)	22 (56.4)	70 (48.6)	0.249
**CNS metastasis**	47 (23)	3 (14.3)	8 (20.5)	36 (25.0)	0.506
**Adrenal metastasis**	26 (12.7)	3 (14.3)	3 (7.7)	20 (13.9)	0.574
**EGFR-TKI**					0.041
**Gefitinib**	51 (25.0)	3 (5.3)	7 (18.4)	41 (10.8)	
**Erlotinib**	77 (37.7)	11 (52.4)	10 (25.6)	56 (38.9)	
**Afatinib**	76 (37.3)	7 (33.3)	22 (56.4)	47 (32.6)	
**Anti-VEGF**					< 0.001
**Bevacizumab**	39 (19.1)	17 (81.0)	22 (56.4)	0 (0)	
**Ramucizumab**	21 (10.3)	4 (19.0)	17 (43.6)	0 (0)	

*CNS* central nervous system, *ECOG PS* Eastern Cooperative Oncology Group performance status, *EGFR* epidermal growth factor receptor, *LN* Lymph Node, *TKI* tyrosine kinase inhibitor, *VEGF* Vascular endothelial growth factor Continuous variables are presented as the mean (standard deviation) or median (interquartile range); categorical variables are presented as the number and percentage

### Survival outcomes and clinical outcome prediction factors for all patients

**Fig. 2:**
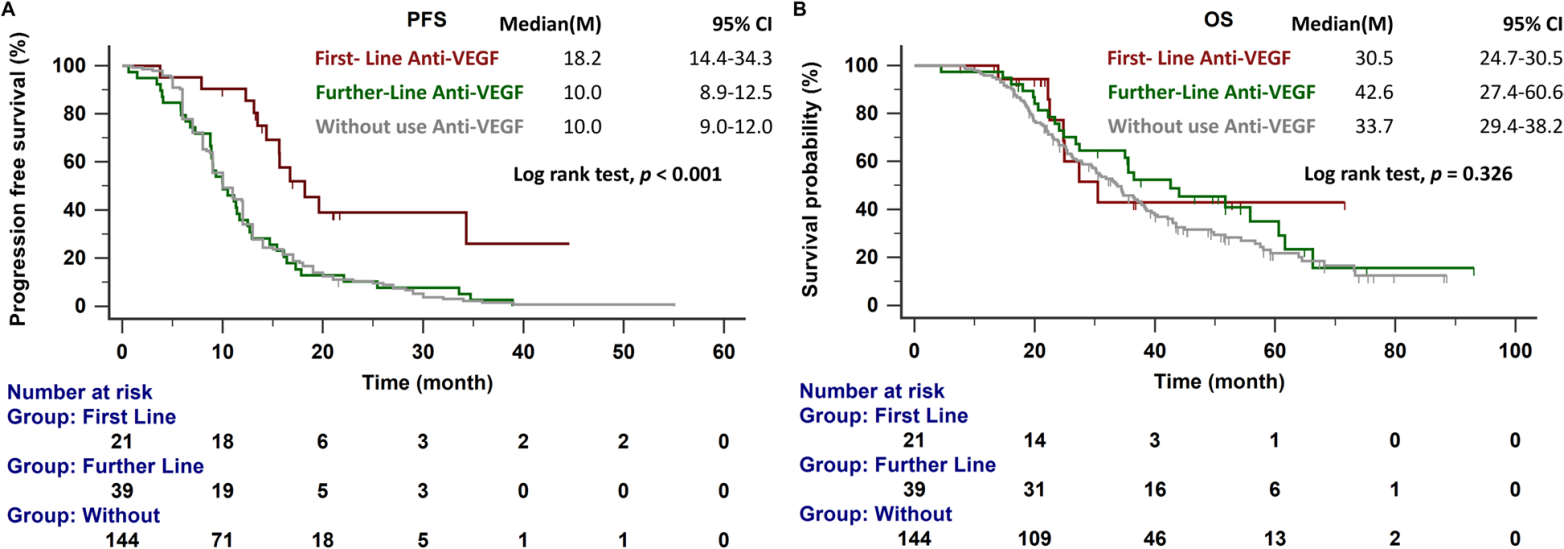
**A** PFS in patients with EGFR-mutant NSCLC treated with first-line, further-line, or no anti-angiogenesis agents. **B** OS in patients with EGFR-mutant NSCLC treated with first-line, further-line, or no anti-angiogenesis agents. EGFR, epidermal growth factor receptor; NSCLC, non–small cell lung cancer; OS, overall survival; PFS, progression-free survival

After a median follow-up of 52.8 months (range 50.5–64.8 months), 136 of 204 patients had died. The median PFS among patients receiving combination therapy with an anti-angiogenesis drug and an EGFR-TKI as first-line therapy was significantly longer than that for patients receiving EGFR-TKI monotherapy (18.2 vs. 10.0 months; *p* < 0.001; Fig. 2A). No significant difference in median OS was observed among patients receiving first-, further-line, or no anti-angiogenesis therapy (30.5 vs. 42.6 vs. 33.7 months, *p* = 0.326; Fig. 2B). Cox proportional hazards regression analysis was used to identify prognostic factors for poor OS, which revealed that ECOG PS ≥ 2 (hazard ratio [HR]: 1.977, 95% confidence interval [CI]: 1.05–3.72), L858R point mutation (HR: 1.431, 95% CI: 1.00–2.05), pleural metastasis (HR: 1.852, 95% CI: 1.28–2.68), liver metastasis (HR: 1.774, 95% CI: 1.13–2.78), and bone metastasis (HR: 1.829, 95% CI: 1.25–2.68) are independent prognostic factors for poor OS (Table 2).

**Table 2:** Univariate and Multivariate Analysis of Clinical Factors Associated with Overall Survival

	Univariate			Multivariate model		
	HR	95% CI	***p***-value	HR	95% CI	***p***-value
**Age ≥ 65 y**	1.562	1.11–2.21	0.013	1.106	0.99–1.03	0.194
**ECOG PS ≥ 2**	2.235	1.25–3.98	0.014	1.977	1.05–3.72	0.035
**Smoking**	1.028	0.67–1.56	0.897	1.028	0.66–1.59	0.901
**L858R vs. Del 19** ***EGFR*** **Mutation**	1.637	1.16–2.31	0.005	1.431	1.00–2.05	0.049
**Pleural Metastasis**	1.437	1.03–2.01	0.034	1.852	1.28–2.68	0.001
**Liver Metastasis**	1.620	1.08–2.42	0.024	1.774	1.13–2.78	0.013
**Bone Metastasis**	1.845	1.31–2.61	< 0.001	1.829	1.25–2.68-	0.002
**CNS Metastasis**	1.107	0.74–1.66	0.620	1.104	0.72–1.69	0.644
**Anti-VEGF**	0.738	0.49–1.10	0.128	0.755	0.48–1.16	0.208

*CI* confidence interval, *CNS* central nervous system, *ECOG PS* Eastern Cooperative Oncology Group performance status, *EGFR* epidermal growth factor receptor, *HR* hazard ratio, *VEGF* vascular endothelial growth factor

### Subgroup analysis of survival outcome

**Fig. 3:**
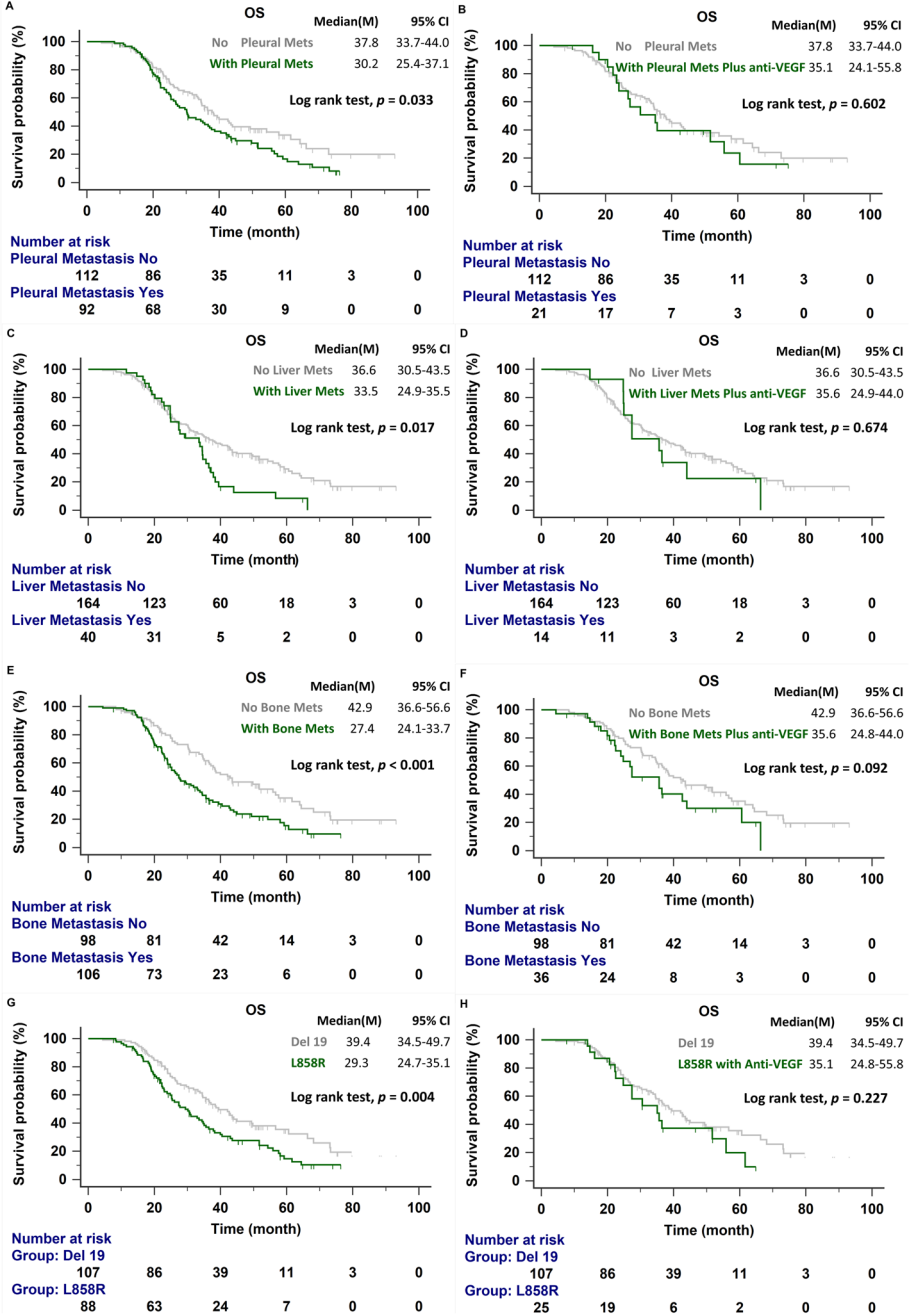
**A** OS in patients with and without pleural metastasis. **B** OS in patients with pleural metastasis treated with anti-angiogenesis drug and patients without pleural metastasis. **C** *OS* in patients with and without liver metastasis. **D** OS in patients with liver metastasis treated with anti-angiogenesis drug and patients without liver metastasis. **E** OS in patients with and without bone metastasis. **F** OS in patients with bone metastasis treated with anti-angiogenesis drug and patients without bone metastasis. **G** OS in patients with exon 19 deletion and exon 21 L858R mutation. **H** OS in patients with exon 19 deletion and exon 21 L858R mutation treated with an anti-angiogenesis drug. OS, overall survival; Mets, metastasis; VEGF, vascular endothelial growth factor

As shown in Table 2, significantly shorter OS was observed among patients with pleural metastasis than among those without pleural metastasis (30.2 vs. 37.8 months, *p* = 0.033; Fig. 3A). Among patients receiving anti-angiogenesis agents at any point in their treatment course, pleural metastasis was no longer a significant prognostic factor (35.1 vs. 37.8 months, p = 0.602; Fig. 3B). A similar effect was observed among patients with and without liver (33.5 vs. 36.6 months, *p* = 0.017, Fig. 3C; with anti-angiogenesis treatment: 35.6 vs. 36.6 months, *p* = 0.674; Fig. 3D) and bone metastasis (27.4 vs. 42.9 months, *p* < 0.001; Fig. 3E; with anti-angiogenesis treatment: 35.5 vs. 42.9 months, *p* = 0.092; Fig. 3F). The median OS was significantly longer in patients with exon 19 deletion than with exon 21 L858R point mutation (39.4 vs. 29.3 months, *p* = 0.004; Fig. 3G). In patients receiving an anti-angiogenesis drug, the median OS for patients with exon 21 L858R point mutation was similar to that for patients with exon 19 deletion (35.1 vs. 39.4 months, *p* = 0.227; Fig. 3H).

### Acquired T790M mutation after disease progression

**Fig. 4:**
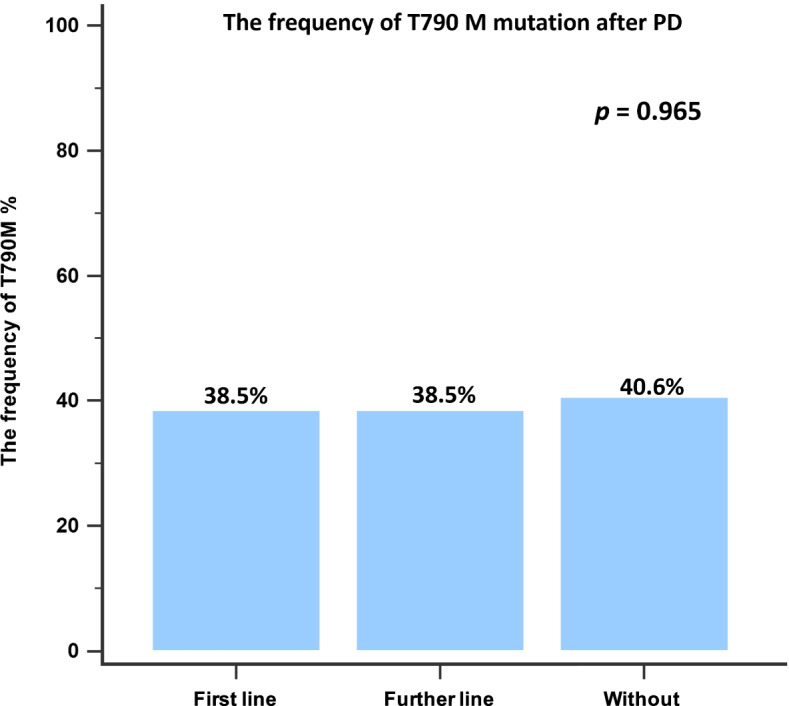
The incidence of T790M mutation development after disease progression among patients treated with first-line, further-line, or no anti-angiogenesis drug. PD, progressive disease

Disease progression occurred in 13 patients (13/21, 61.9%) who received first-line anti-angiogenesis therapy, 38 patients (39/39, 100%) who received further-line anti-angiogenesis therapy, and 143 patients (143/144, 99.3%) who received no anti-angiogenesis therapy. All patients who experienced progression underwent re-biopsy, either liquid or tissue biopsy, and the presence of the T790M mutation was evaluated. Among patients who received first-line combination therapy with an EGFR-TKI and an anti-angiogenesis drug, 5 (5/13, 38.5%) patients were positive for the T790M mutation. No significant difference in the T790M positivity rate was observed among these three groups (5/13, 38.5% vs. 15/39, 38.5% vs. 58/143, 40.6%; *p* = 0.965; Fig. 4).

### Adverse events

The selected toxicity profile is summarized in Table 3. Overall, the incidence of adverse events was comparable in both groups. The most common EGFR-TKI–related adverse events were dermatitis acneiform and paronychia, whereas the most common anti-angiogenesis–related adverse events were proteinuria (21.7%) and hypertension (18.3%). Severe dermatitis acneiform (≥grade 3) was more commonly observed in the group receiving anti-angiogenesis therapy than in the group receiving EGFR-TKI alone. Among the 7 patients with severe dermatitis acneiform, 4 patients received first-line EGFR-TKI and anti-angiogenesis combination therapy. Among the severe adverse events leading to permanent discontinuation of anti-angiogenesis therapy, one patient who received ramucirumab discontinued therapy due to severe hypertension, one patient who received bevacizumab discontinued therapy due to severe nasal bleeding, and one patient who received afatinib discontinued therapy due to progressive interstitial lung disease (Table 3). No treatment-related deaths occurred in this study.

**Table 3:** Selected toxicity profile

	With anti-angiogenesis (***n*** = 60)		Without anti-angiogenesis (***n*** = 144)	
	Any grade	≥ grade 3	Any grade	≥ grade 3
Hepatitis	10 (16.7%)	0 (0%)	18 (12.5%)	0 (0%)
Diarrhea	26 (43.3%)	1 (1.7%)	52 (36.1%)	1 (0.7%)
Dermatitis acneiform	35 (58.3%)	7 (11.7%)	56 (38.9%)	4 (2.8%)
Paronychia	29 (48.3%)	1 (1.7%)	60 (41.7%)	5 (3.5%)
Skin rash	6 (10%)	3 (5%)	27 (18.8%)	6 (4.2%)
Oral ulcer	8 (13.3%)	0 (0%)	33 (22.9%)	1 (0.7%)
Proteinuria	13 (21.7%)	0 (0%)	0 (0%)	0 (0%)
Hypertension	11 (18.3%)	2 (3.3%)^a^	0 (0%)	0 (0%)
Bleeding	7 (11.7%)	1 (1.7%)^b^	2 (1.4%)	0 (0%)
Interstitial lung disease	1 (1.7%)	1 (1.7%)^c^	0 (0%)	0 (0%)

^a^One patient received Ramucirumab and it was switched to Bevacizumab due to severe hypertension

^b^The patient received Bevacizumab but it was discontinued due to severe nasal bleeding

^c^The patient received Afatinib but it was discontinued due to progressive interstitial lung disease

## Discussion

To the best of our knowledge, this study is the first real-world study examining the effects of combination first- or second-generation EGFR-TKI plus bevacizumab or ramucirumab therapy on PFS compared with EGFR-TKI monotherapy as first-line treatment, which patients should be treated with the combination treatment, and the optimal timing of combination treatment. Combination therapy with an anti-angiogenesis drug appears to provide a survival benefit, regardless of whether the anti-angiogenesis agent is used as first- or further-line therapy, in patients with EGFR-mutant NSCLC harboring L858R mutation who experience pleural, liver, or bone metastasis. The frequency of T790M mutation acquisition after EGFR-TKI therapy combined with either a first- or further-line anti-angiogenesis agent was similar to the frequency observed for EGFR-TKI monotherapy. Based on these results, a clinician could add anti-angiogenesis therapy based on the patient’s general condition and needs, without concern for specific timing.

First-generation EGFR-TKIs combined with bevacizumab or ramucirumab resulted in significantly longer median PFS than EGFR-TKI monotherapy (16–19 months vs. 9–13 months) in untreated patients with advanced-stage EGFR-mutant NSCLC harboring either the exon 19 deletion or the L858R mutation in a phase II/III trial [13–15]. A multicenter real-world study demonstrated that the use of a second-generation EGFR-TKI plus bevacizumab provided a longer median PFS (23.9 months) [16]. Huang et al. also indicated that the use of a first- or second-generation EGFR-TKI plus bevacizumab resulted in longer PFS (17.1 vs. 21.6 months; *p* = 0.617) [17]. However, these two studies did not include an EGFR-TKI monotherapy group for comparison. A real-world study with propensity score matching (PSM) from China indicated that first-generation EGFR-TKIs plus bevacizumab could lead to the significant prolongation of PFS compared with EGFR-TKI monotherapy (16.5 vs. 12.0 months; *p* = 0.001) [23]. Another PSM study from Taiwan showed that bevacizumab plus EFGR-TKIs improved PFS compared with EGFR-TKI alone (17.0 vs. 11.0 months; *p* = 0.002) [21]. However, the former study did not include patients receiving second-generation EGFR-TKIs, and the latter study did not include patients receiving ramucirumab. The current study included the use of both first- and second-generation EGFR-TKIs combined with multiple anti-angiogenesis drugs in the final analysis, which revealed that the median PFS of patients treated with an EGFR-TKI and an anti-angiogenesis drug as first-line therapy was longer than the PFS of patients treated with EGFR-TKI monotherapy (18.2 vs. 10.0 months). The PFS of patients receiving first-line combination therapy with an anti-angiogenesis drug plus an EGFR-TKI was comparable to the PFS reported for previous randomized controlled trials.

Adding an anti-angiogenesis drug to EGFR-TKI as first-line therapy did not improve the OS significantly in our cohort, which was consistent with previous studies [13, 14]. The inclusion of anti-angiogenesis therapy in later-line therapy regimens had a better effect in patients with advanced NSCLC, resulting in longer PFS-2, [18, 19] which has previously been positively correlated with improved OS [20]. Therefore, the inclusion of anti-angiogenesis therapy in later-line regimens may remain a crucial contributor to prolonged survival. Clinicians should identify candidates who are likely to receive a survival benefit from combination therapy at any line of treatment. Tsai et al. showed that combination therapy using an EGFR-TKI and bevacizumab provides better OS than the use of an EGFR-TKI alone in patients harboring the L858R mutation [21]. Exon 19 deletion and exon 21 L858R point mutation are regarded as two separate entities, resulting in different structural changes in EGFR, differences in the rate of concomitant mutations, and differences in the overall tumor mutation burden [24]. Survival outcomes have been poor among the L858R patient groups in various EGFR-TKI monotherapy studies [1–6]. In the present study, the median OS was also significantly shorter among patients with the L858R mutation than among patients with the exon 19 deletion. The combination therapy strategy may provide clinical benefits for those with the L858R mutation. In the NEJ026 and RELAY studies, subgroup analyses demonstrated that erlotinib plus an anti-angiogenesis agent improved PFS in patients with the L858R mutation [14, 15]. The current study showed that the median OS in patients with the L858R mutation was prolonged to 35.1 months after the addition of an anti-angiogenesis agent, and no significant difference in OS was observed between those with exon 19 deletion and those with the exon 21 L858R point mutation (39.4 vs. 35.1 months; *p* = 0.227).

Serum VEGF may be a potential biomarker for predicting the subgroup of patients who may benefit from EGFR-TKIs plus bevacizumab [25]. High VEGF expression has been correlated with the incidence of metastasis and poor prognosis in various cancers [26, 27]. In our cohort, Cox regression analysis revealed that pleural metastasis, liver metastasis, and bone metastasis were independent prognostic factors for poor survival. The addition of an anti-angiogenesis agent, in either first-line or further-line settings, improved OS in patients with poor prognosis (Fig. 3).

Malignant pleural effusion (MPE) is a common complication observed in patients with NSCLC, associated with decreased survival [28]. VEGF has been found to promote MPE development in patients with NSCLC through increased vascular permeability and the promotion of angiogenesis [29, 30]. Anti-angiogenesis was hypothesized to play a potential role in MPE management. Bevacizumab was previously demonstrated to provide therapeutic benefits for patients with NSCLC and MPE [31, 32]. High expression of VEGF is also associated with liver metastasis and poor clinical outcomes in patients with pancreatic adenocarcinoma [33]. Liver metastasis has been found to be the worst prognostic factor for metastatic lung adenocarcinoma, and the addition of bevacizumab treatment might improve prognosis [34, 35]. Bone is one of the most common distal metastatic sites in patients with lung adenocarcinoma, and bone metastasis is associated with significant morbidity and metabolic disorders, such as hypercalcemia, pathologic fractures, and spinal cord compression, which result in poor prognosis [36]. A few studies have reported high expression levels of VEGF and its receptors in bone metastases from primary human breast cancer or hepatocellular carcinoma [37, 38]. Therefore, VEGF signaling may be a therapeutic target for osteoclast inhibition. Hu et al. indicated that radiotherapy combined with targeted therapy resulted in the strong inhibition of cyclooxygenase 2 and VEGF expression in bone metastasis from lung cancer, which improved efficacy and prolonged survival [39]. The current study indicated that pleural, liver, and bone metastases are independent factors for mortality. However, among patients receiving an anti-angiogenesis during the treatment course, no significant difference in the median OS was observed between groups with and without pleural, liver, and bone metastases.

The frequency of the T790M mutation should be evaluated after progression on EGFR-TKI and anti-angiogenesis treatment because osimertinib is used as a further-line treatment to prolong survival in patients who are T790M positive. The rates of T790M mutation in the RELAY study were 43 and 47% in the ramucirumab plus erlotinib group and the erlotinib alone group, respectively. Tsai et al. reported similar T790M mutation frequencies after treatment failure for both combination treatment and EGFR-TKI monotherapy (46.7% vs. 53.6%). The current study also showed a similar incidence of T790M mutation acquisition among the three groups (38.5% vs. 38.5% vs. 40.6%; *p* = 0.965).

Combination therapy consisting of an anti-angiogenesis drug and an EGFR-TKI increased the incidence of adverse events of any grade compared with EGFR-TKI therapy alone, especially proteinuria (21.7%), hypertension (18.3%), and bleeding (11.7%), consistent with previous real-world studies [17, 21]. These incidence rates were lower than those recorded in clinical trials, [14, 15] likely due to the lower dose of bevacizumab (7.5 mg/kg) used in our study. Based on these results, combination therapy is an effective and safe treatment for patients with *EGFR*-mutant NSCLC.

Our study had some limitations. First, this was a retrospective, single-institution study, and the number of patients in our cohort was limited. However, this represents a real-world study evaluating the efficacy of anti-angiogenesis drugs, such as bevacizumab and ramucirumab, in combination with both first- and second-generation EGFR-TKIs. Our study is the first to include ramucirumab in a real-world setting, but further analysis was limited due to small patient numbers. Details on the prognostic effects of different metastatic sites were also evaluated, which have not been described in previous studies. This study indicated which patients were likely to derive an OS benefit from combination EGFR-TKI and anti-angiogenesis treatment. Second, we only included patients who received either tissue or liquid re-biopsy to determine the acquisition of the T790M mutation in our final analysis, which may have resulted in selection bias; therefore, a multivariate analysis was performed to minimize selection bias. Finally, unlike a previous study that included patients with advanced-stage NSCLC, we only included patients with stage IV NSCLC to enable the analysis of metastatic patterns as prognostic predictors of survival with and without the use of an anti-angiogenesis drug.

## Conclusion

In summary, our study revealed that upfront treatment with an anti-angiogenesis drug combined with an EGFR-TKI provided better PFS than the use of an EGFR-TKI alone as first-line treatment. In patients harboring the L858R mutation or those with pleural, liver, or bone metastases, adding an anti-angiogenesis drug at any time during the treatment course, in either first- or further-line settings, improves the survival probability.

## Data Availability

All data generated or analyzed during this study are included in this published article.

## Abbreviations

CT: Computed tomography
Del19: EGFR exon 19 deletion
ECOG PS: Eastern Cooperative Oncology Group performance status
EGFR: Epidermal growth factor receptor
EGFR-TKI: Epidermal growth factor receptor tyrosine kinase inhibitor
IQR: Interquartile range
MPE: Malignant pleural effusion
MRI: Magnetic resonance imaging
NSCLC: Non–small cell lung cancer
ORR: Objective response rate
OS: Overall survival
PET: Positron emission tomography
PFS: Progression-free survival
TKI: Tyrosine kinase inhibitor
VEGF: Vascular endothelial growth factor
VEGFR: Vascular endothelial growth factor receptor
